# Nitric oxide regulates perylenequinones biosynthesis in *Shiraia bambusicola* S4201 induced by hydrogen peroxide

**DOI:** 10.1038/s41598-021-81990-2

**Published:** 2021-01-27

**Authors:** Ning Zhao, Yingying Yu, Yunxia Yue, Mingzhu Dou, Bingjing Guo, Shuzhen Yan, Shuanglin Chen

**Affiliations:** 1 College of Life Sciences, Nanjing Normal University, No.1 Wenyuan Road, Qixia District, Nanjing, 210023 People’s Republic of China; 2 Microbiology Laboratory, Nanjing Municipal Center for Disease Control and Prevention, Nanjing, 210003 People’s Republic of China

**Keywords:** Fungal physiology, Fungal genes, Microbiology

## Abstract

*Shiraia bambusicola* has been used as a traditional Chinese medicine for a long history. Its major medicinal active metabolites are perylenequinones, including hypocrellin A, elsinochrome A and so on. At present, the fermentation yield of perylenequinones is low, and its complex biosynthesis and regulatory pathways are still unclear. In this study, nitric oxide, as a downstream signal molecule of hydrogen peroxide, regulates the biosynthesis of perylenequinones. Exogenous addition of 0.01 mM sodium nitroprusside (nitric oxide donor) can promote perylenequinones production by 156% compared with the control. Further research found that hydrogen peroxide and nitric oxide increased the transcriptional level of the biosynthetic genes of hypocrellin A. The results showed that nitric oxide is involved in the biosynthesis and regulation of perylenequinones in *Shiraia bambusicola* as a signal molecule. In the future, the yield of perylenequinones can be increased by adding exogenous nitric oxide in fermentation.

## Introduction

*Shiraia bambusicola*is a rare parasitic fungus belonging to Ascomycota, which is also a traditional medicinal resource in China. This fungus usually parasitizes on the twigs of *Brachystachyum* and other bamboos, and the fruiting body is known as *S. bambusicola*. *S. bambusicola* is mainly distributed in the south of the Yangtze River in China, as well as in Japan^1^. *S. bambusicola* can be used to treat pertussis, sciatica, tracheitis and other diseases. The mainly bioactive secondary metabolites in *S. bambusicola* are hypocrellins (hypocrellin A, hypocrellin B, hypocrellin C, hypocrellin D) which belongs to perylenequinones (PQs). As a photosensitizer, hypocrellin A (HA) has the ability of antibacterial, antiviral, antitumor and so on, because it can be activated under light irradiation and can produce reactive oxygen species (ROS) which can destroy cells^2–4^. However, the low yield of HA from fruiting bodies or traditional fermentations limits its application. Many studies have been devoted to increasing the yield of HA by adding different reagents or changing fermentation conditions, such as using Triton X-100^5^, adding Ca^2+^^6^, changing light conditions^7^ and so on.

There are many kinds of PQs, most of which exist in plant pathogenic fungi, including cercosporin, elsinochromes, hypocrellins, hypericin and so on^8^. They are similar in structure and have similar biosynthetic pathways^9, 10^. According to the biosynthetic pathway of cercosporin and elsinochrome C, Zhao et al. proposed a putative biosynthetic pathway of HA^11^. Acetyl-CoA and malonyl-CoA were used as substrates to catalyze the formation of intermediate metabolite *nor*-toralactone by polyketide synthase (PKS). After that, HA may be formed by methylation, hydroxylation, dimerization and redox under the action of O-methyltransferase/FAD-dependent monooxygenase (OF), O-methyltransferase (Omef), FAD/FMN-dependent oxidoreductase (FF) and other enzymes. These enzymes play an important role in HA biosynthetic pathways, the transcriptional level of their encoding genes is closely related to the yield of HA.

ROS are well-known as a normal metabolite of oxygen in aerobically growing cells, providing vital signaling functions for diverse cellular processes^12–16^. As a member of ROS, hydrogen peroxide (H_2_O_2_) acts as a major redox metabolite operative in signaling, redox sensing and redox regulation^17^. Meanwhile, current evidences are accumulating that H_2_O_2_ is also associated with the production of secondary metabolites^18–20^. For example, the concentrations of both anthocyanin and total flavonoids were significantly induced by H_2_O_2_ treatment in *Arabidopsis*^21^. Toxigenic isolate of *Aspergillus flavus* responses to oxidative stress are related to their ability to produce aflatoxin^22^. There is a relationship between ROS and lovastatin biosynthesis in *Aspergillus terreus*^23^. Recently, it was reported that H_2_O_2_ is involved in the biosynthesis of PQs. When H_2_O_2_ at 30 µM was applied, the HA production of *Shiraia* sp. A8 was most effectively enhanced^24^. And hypocrellin production of *Shiraia* sp. SUPER-H168 was significantly improved when treated with high concentrations of H_2_O_2_ (10–20 mM)^25^. These evidences suggest that H_2_O_2_ may be involved in the biosynthesis of PQs, but the signal relationship or regulatory pathway is not clear.

Nitric oxide (NO) is a critical signaling molecule, which plays a broad role in regulating organisms physiological and biochemical functions^26^. It is now clear that H_2_O_2_ and NO function as signaling molecules in plants. Cellular responses to H_2_O_2_ and NO are complex, with considerable cross-talk between responses to several stimuli^27^. NO depends on the production of H_2_O_2_ and salicylic acid, which mediates the fungus-induced effects on the accumulation of volatile oil in *Atractylodes lancea* plantlets. These three signal molecules play a key role in regulating the accumulation of volatile oil production induced by the fungus^28^. The endophytic fungus *Phomopsis liquidambari* increases nodulation and N_2_ fixation in *Arachis hypogaea* by enhancing H_2_O_2_ and NO signaling, and H_2_O_2_ may act upstream of NO^29^. Liu et al. reported that NO functions in signaling and has a close relationship with Ca^2+^ in heat stress—induced ganoderic acid biosynthesis^30^. These evidences suggest that NO may be involved in the biosynthesis of secondary metabolites.

In this study, H_2_O_2_ treatment was applied to increase the cytosolic NO content. As the downstream signal molecule of H_2_O_2_, NO is able to increase the yield of PQs. In addition, it was found that the increase of PQs production was mainly due to the increase of HA and EA production. Since that H_2_O_2_ and NO did not affect PQs production by chemical contact, the transcriptional level of HA biosynthetic genes was evaluated. The results indicated that NO increased HA production mainly by promoting transcriptional level of HA biosynthetic genes. This research found that exogenous addition of NO can increase the PQs production, which provides a theoretical reference for increasing the yield of secondary metabolites of *S. bambusicola* and other fungal species.

## Materials and methods

### Experimental strain and growth conditions

*S. bambusicola* S4201 was isolated from the fruiting bodies of *S. bambusicola* in China, a strain which has shown excellent HA production. The strain S4201 was cultured on potato dextrose agar (PDA, 200 g/L potato extract, 20 g/L glucose, 20 g/L agar) medium. The spores of S4201 cultured for 120 h were washed with sterile water and prepared into a spore suspension with a final concentration of 1 × 10^6^ spores/mL. Then, S4201 was incubated in potato dextrose broth (PDB, 200 g/L potato extract, 20 g/L glucose, pH 7.0) medium with Triton X-100 for 120 h at 28 °C on a shaker at 150 rpm.

### Chemicals and treatments

H_2_O_2_ solution was used as H_2_O_2_ donor and catalase (CAT) as the scavenger. Sodium nitroprusside (SNP) was used as the NO donor and 2-(4-carboxyphenyl)-4,4,5,5-tetramethylimidazoline-1-oxyl-3-oxide potassium salt was the specific scavenger. H_2_O_2_, CAT, SNP and cPTIO were purchased from Sigma-Aldrich (St. Louis, MO, USA). The concentration of H_2_O_2_ was set to 0.005, 0.01, 0.05, 0.1, 0.5, 1, 2, 4 mM; the concentration of CAT was set to 10, 50, 250, 1250 U/L; the concentration of SNP was set to 0.001, 0.005, 0.01, 0.05, 0.1, 0.5 and 1 mM; the concentration of cPTIO was 0.05 mM. All the reagents were dissolved and diluted with sterile distilled water and filtered through 0.22 μm diameter sterile filters. The same amount of sterile distilled water was used for control^31^. In order to explore the relationship between H_2_O_2_ and NO, exogenous reagents were used for verification. CAT, cPTIO, H_2_O_2_, CAT + H_2_O_2_, cPTIO + H_2_O_2_, SNP, CAT + SNP and cPTIO + SNP were added to the medium for 80 h at 28 °C. The biomass, sugar content and PQs yield were detected to determine the relationship between H_2_O_2_ and NO in PQs biosynthesis.

### Detection of biomass and sugar content

After the liquid fermentation of *S. bambusicola* S4201 with different treatments were finished, the mycelia were filtered and dried to constant weight at 55 °C and weighed. Anthrone-sulfuric acid colorimetric method was used to determine the soluble sugar content in liquid fermentation broth of *S. bambusicola* S4201 treated with different treatments^32^. The standard curve was established as follows: glucose solutions with concentration of 0.1, 0.2, 0.3, 0.4, 0.5, 0.6, 0.7, 0.8, 0.9 and 1 mg/mL were configured. The detection of glucose concentration was carried out by using microplate reader. 625 nm was used as the detection wavelength. The linear regression equation of soluble sugar was obtained by the optical density and concentration.

### Determination of PQs production

In order to detect the production of PQs in the secondary metabolites of *S. bambusicola* S4201, a linear relationship between the optical density and the concentration of PQs was established^11^. The detection of PQs was carried out by microplate reader (SpectraMax M2). 464 nm was used as the detection wavelength. Based on the linear regression of the optical density and concentration, the linear regression equation of HA was obtained. The yield of PQs was calculated with HA production as the standard.

### Extraction and measurement of H_2_O_2_ and NO

The fermentation liquid of *S. bambusicola* S4201 was filtered to obtain mycelium, and liquid nitrogen was used to grind the mycelia into powder. The powdered sample was transferred to a 1.5 mL centrifugal tube, and 400 μL pre-cooled phosphate buffer saline (PBS) was added. The mixture was centrifuged at 4000 rpm for 15 min at 4 °C and the supernatant was used for the detection of protein concentration, H_2_O_2_ and NO. The contents of protein, H_2_O_2_ and NO were measured by Modified Bradford Protein Assay Kit (Sangon Biotech, China), Hydrogen Peroxide assay kit and Nitric Oxide (NO) assay kit (Microwell plate method) (Nanjing Jiancheng Bioengineering Institute, China) according to the manufacturer’s instructions.

### Determination of HPLC

The supernatant of liquid fermentation was extracted with ethyl acetate until it turned colorless. The extracts were concentrated to dryness and the residue was dissolved in acetonitrile. The concentrations of PQs were measured by high-performance liquid chromatography (HPLC) with standard reagents^11^. Agilent Technologies 1220 Infinity LC instrument was used to perform HPLC. The PQs component of *S. bambusicola* S4201 were analyzed by HPLC at 30 °C using a C18 column (5 µm, 4.6 × 250 mm) (SunFire, Waters, USA), with a flow rate of 1 mL/min and injection volume of 10 µL.

### Quantitative real-time PCR analysis

Real-time fluorescence quantitative polymerase chain reaction (qRT-PCR) was used to detect the effect of H_2_O_2_ and NO on HA biosynthetic genes^33^. RNA from different treatment samples were extracted by TRIzol reagent according to the instructions of the manufacturer (Vazyme, Nanjing, China) and cDNA were synthesized using HiScript II 1st Strand cDNA Synthesis Kit (Vazyme, Nanjing, China). qRT-PCR was performed using a StepOnePlus Real-Time PCR System (Applied Biosystems, USA) and AceQ qPCR SYBR Green Master Mix (Vazyme, Nanjing, China). Glyceraldehyde-3-phosphate dehydrogenase (GAPDH) gene was used as internal reference and the primers were designed by Beacon Designer8. The primer sequences are shown in Table S1. The relative expression level of the genes were calculated by 2^−ΔΔCt^ method^34^. All the experimental data were obtained from three biological replicates.

### Statistical analyses

All statistical analyses were performed using SPSS 16.0 (SPSS Inc., Chicago, IL, USA). Independent t-test was used to analyze the data when there was only one control group and one experimental group. Duncan's multiple-range test of one-way ANOVA was used to statistically analyze the significant difference between the multiple samples (*p* < 0.05), when three or more treatment groups were compared. The mean values from the individual experiments were expressed as means ± standard deviations (SE). Five replicates were assessed for each treatment.

### Accession number

Strain S4201 was deposited at China General Microbiological Culture Collection Center (CGMCC) under the accession CGMCC NO. 15864.

### Ethical approval

No human participants or animals were used in this study.

## Results

### H_2_O_2_ increased the PQs production

**Figure 1:**
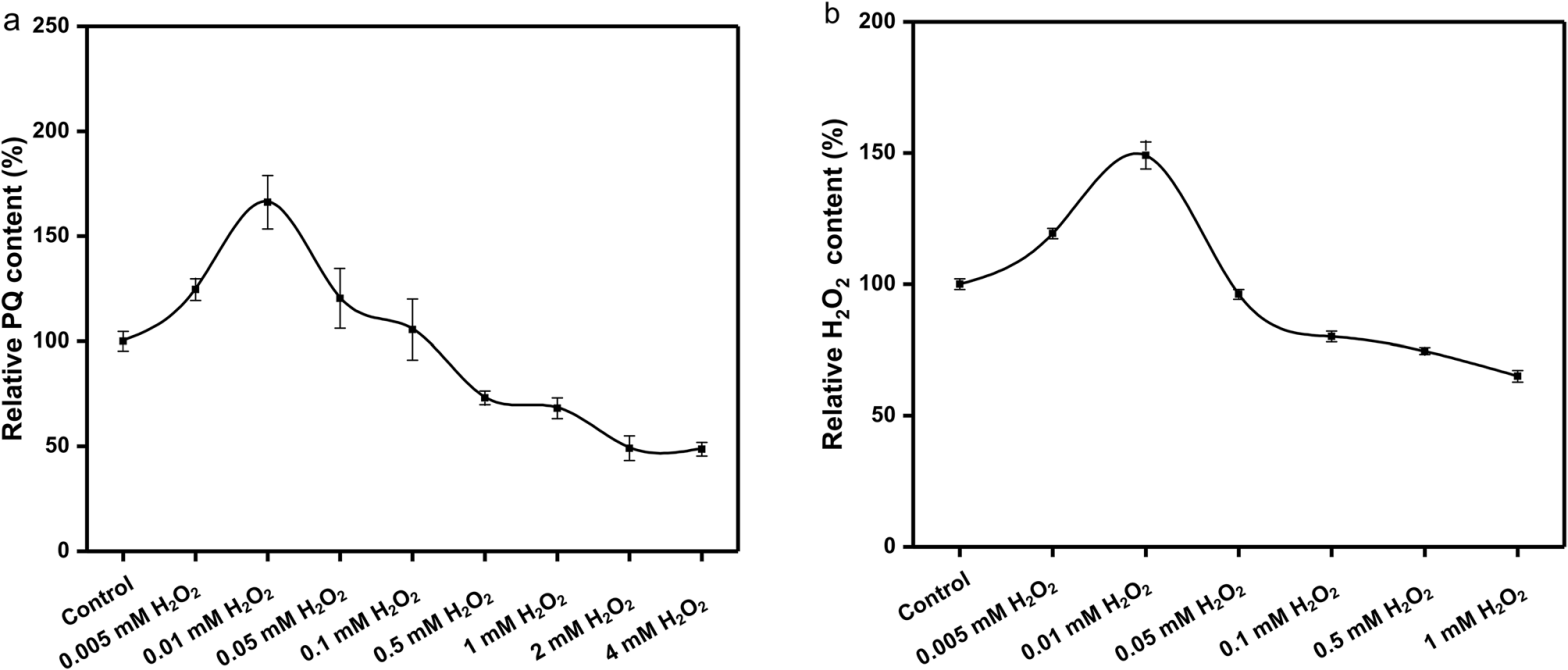
Effects of different concentrations of H_2_O_2_ on perylenequinones production and intracellular H_2_O_2_. Values are the means of five independent experiments ± SE.

In order to study the effect of H_2_O_2_ on accumulation of PQs in *S. bambusicola* and select the appropriate concentration, different concentrations of H_2_O_2_ and CAT were added respectively to liquid culture. When 0.01 mM H_2_O_2_ was added, the content of PQs (the linear regression equation for the y = 0.0092x + 0.1271, R^2^ = 0.9934) and intracellular H_2_O_2_ reached the maximum. When the H_2_O_2_ concentration reached 0.05 mM, the content of PQs and intracellular H_2_O_2_ were significantly decreased (Fig. 1). The promotion of PQs accumulation by H_2_O_2_ can be inhibited by its specific scavenger CAT. Under the action of 250 U/L CAT, the accumulation of PQs content was significantly inhibited. The results showed that under the condition of 28 °C for 80 h, low H_2_O_2_ concentration can promote PQs yield whereas high H_2_O_2_ concentration decreases PQs yield. Therefore, the concentrations of 0.01 mM H_2_O_2_ and 250 U/L CAT were selected for the follow-up experiments.

### NO promoted the PQs production induced by H_2_O_2_

**Figure 2:**
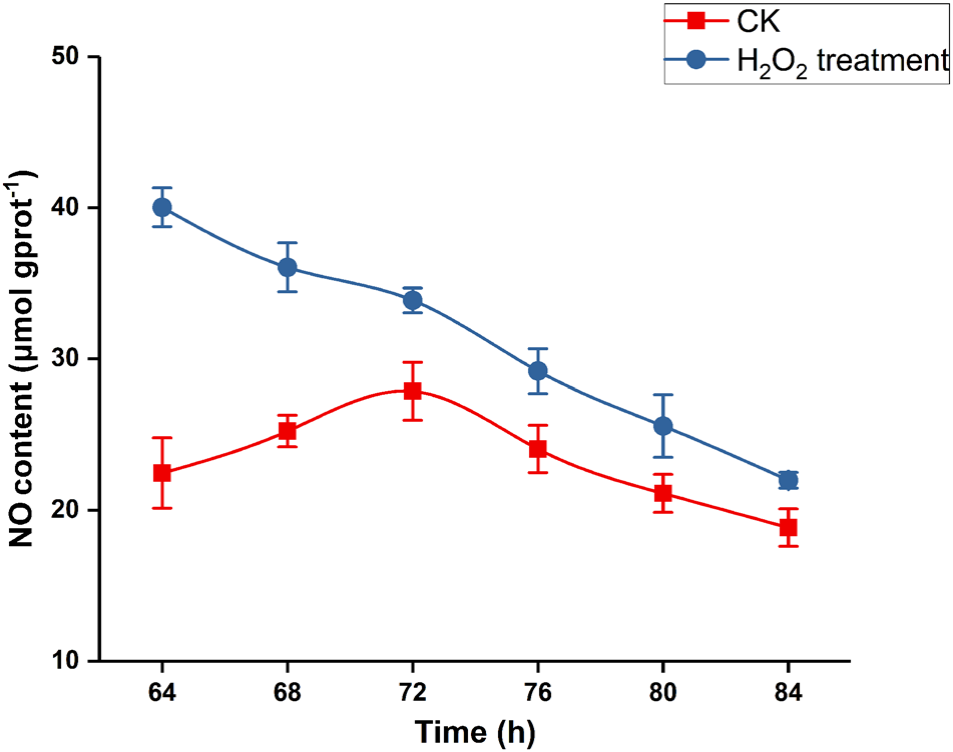
Effects of exogenous hydrogen peroxide on the content of intracellular nitric oxide. Values are the means of five independent experiments ± SE.

**Figure 3:**
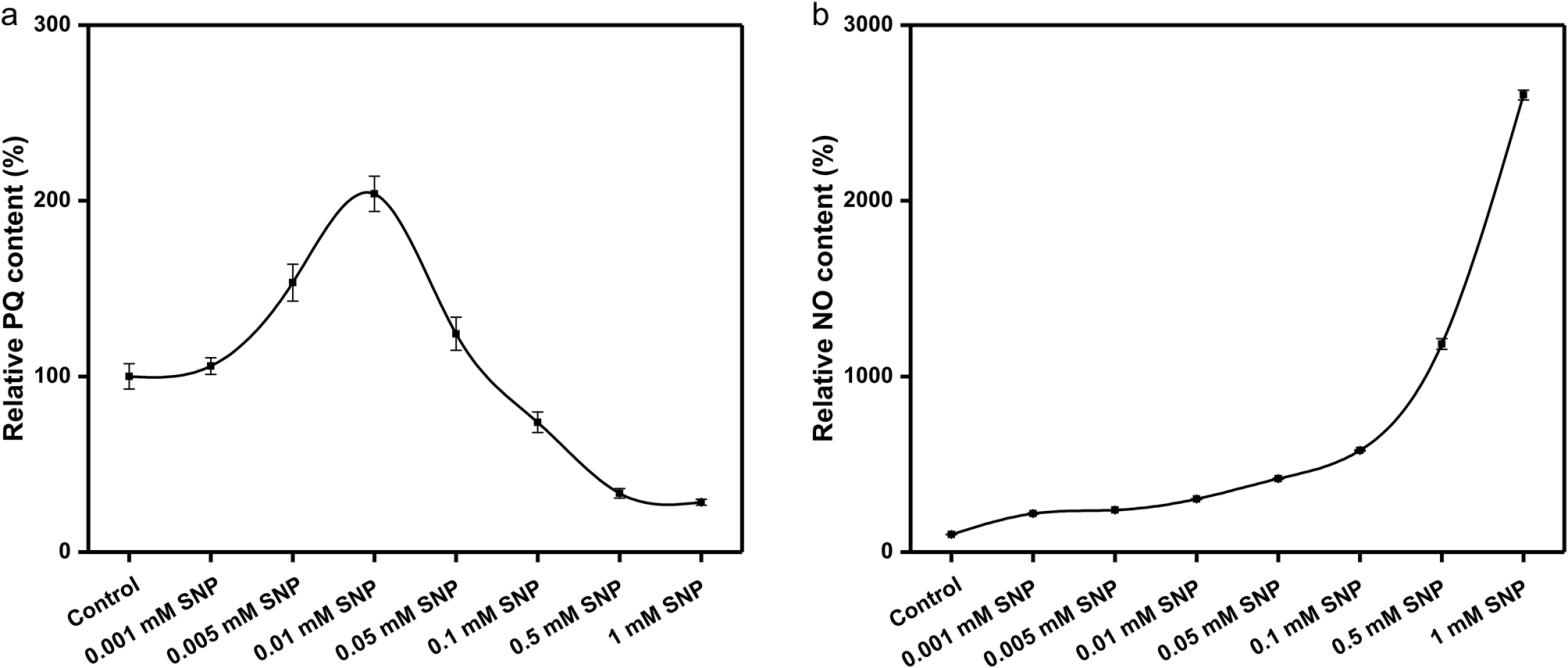
Effects of different concentrations of SNP on perylenequinones production and intracellular NO. Values are the means of five independent experiments ± SE.

H_2_O_2_ and NO are often involved in the production of secondary metabolites as signal molecules in plants. The level of cytosolic NO was analyzed in 64 h-old, 68 h-old, 72 h-old, 76 h-old, 80 h-old and 84 h-old strains grown at 28 °C. Higher amount of NO were observed after H_2_O_2_ treatments than that of control. After adding H_2_O_2_ for 64 h, 68 h, 72 h 80 h and 84 h, the concentration of intracellular NO was 78%, 42%, 22%, 21%, 21% and 17% higher than that of the control, respectively (Fig. 2). These results indicate that H_2_O_2_ treatment can induce an increase of cytosolic NO content. In order to study the effect of NO on the content of PQs, different concentrations of SNP and cPTIO were added respectively for liquid fermentation. When 0.01 mM SNP was added, the content of PQs reached the maximum (Fig. 3). And the promoting effect of SNP on PQs biosynthesis can be inhibited by its specific scavenger cPTIO. When 0.05 mM cPTIO was added, the yield of PQs could be significantly inhibited. Therefore, the concentrations of 0.01 mM SNP and 0.05 mM cPTIO were selected for the follow-up experiments.

### NO act as downstream signal molecule of H_2_O_2_

**Figure 4:**
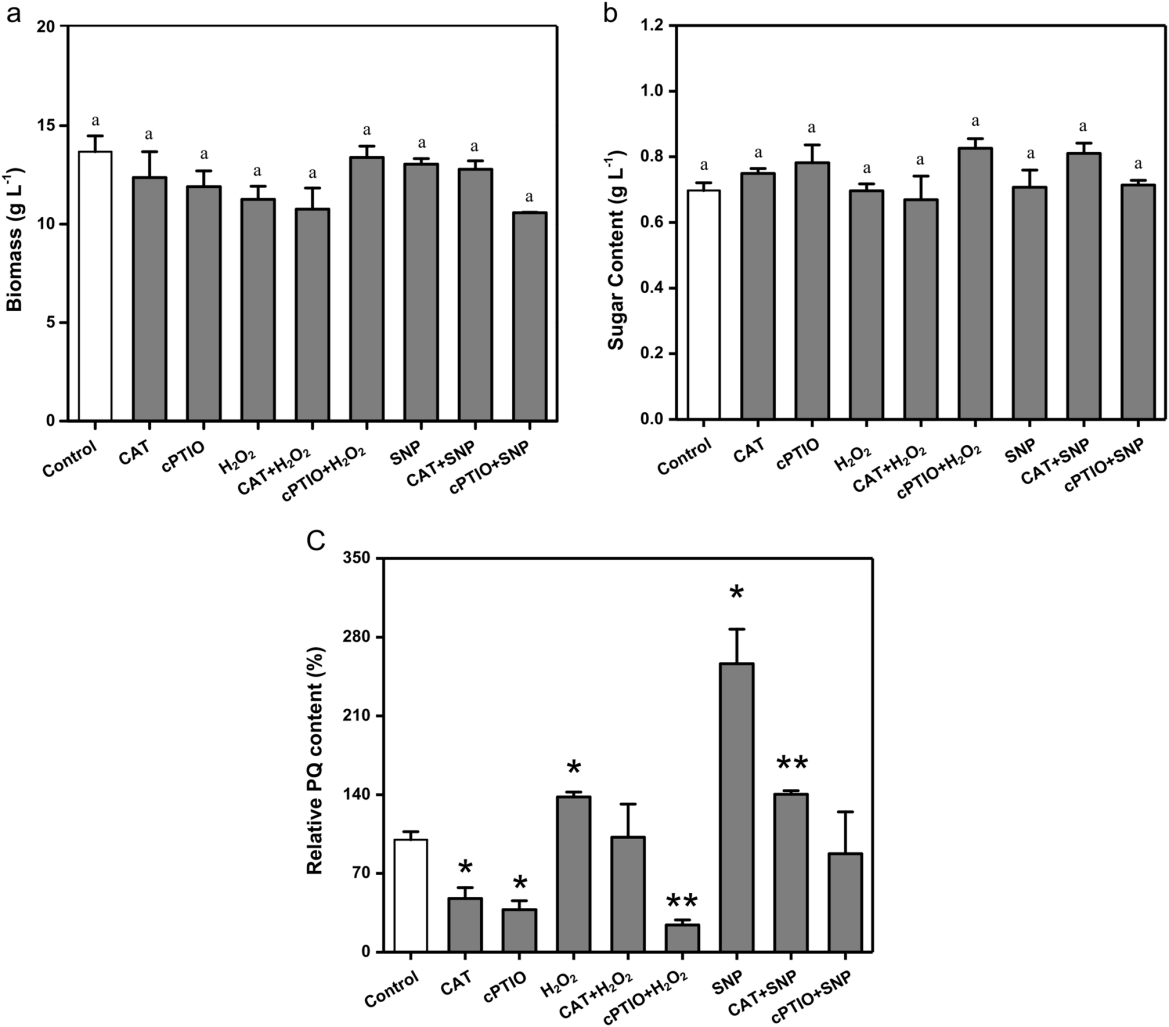
Interactions between H_2_O_2_ and NO signaling pathways in *Shiraia bambusicola* S4201. (**a**) Biomass; (**b**) Sugar content; (**c**) Perylenequinones content. Values are the means of five independent experiments ± SE. Different letters and asterisks on the histogram indicate significant differences between treatments (*p* < 0.05).

Different exogenous addition treatment can help to verify the relationship of H_2_O_2_ and NO in the progress of promoting PQs yield. As shown in Fig. 4, the biomass and sugar content (linear regression equation for the y = 2.6733x + 0.2083, R^2^ = 0.9938) of all different treatment groups were not significantly different from that of the control group, indicating that H_2_O_2_, NO and their scavengers had no significant effects on mycelial growth and energy consumption. Meanwhile, both H_2_O_2_ and NO significantly increased the yield of PQs (1.38-fold and 2.56-fold), while their scavengers significantly decreased the yield of PQs. On the basis of CAT and cPTIO, the yield of PQs could be restored to a level where there was no significant difference compared with the control group by adding H_2_O_2_ and SNP, respectively. In the treatment of cPTIO + H_2_O_2_, cPTIO could significantly inhibit the increase of PQs induced by H_2_O_2_, while in treatment of CAT + SNP, CAT could not significantly inhibit the increase of PQs content induced by SNP. These results suggest that H_2_O_2_ and NO are closely related in the progress of increasing PQs yield, and NO may act as the downstream signal molecule of H_2_O_2_.

### H_2_O_2_ and NO induced biosynthesis of PQs by promoting the transcriptional level of the biosynthetic genes

**Figure 5:**
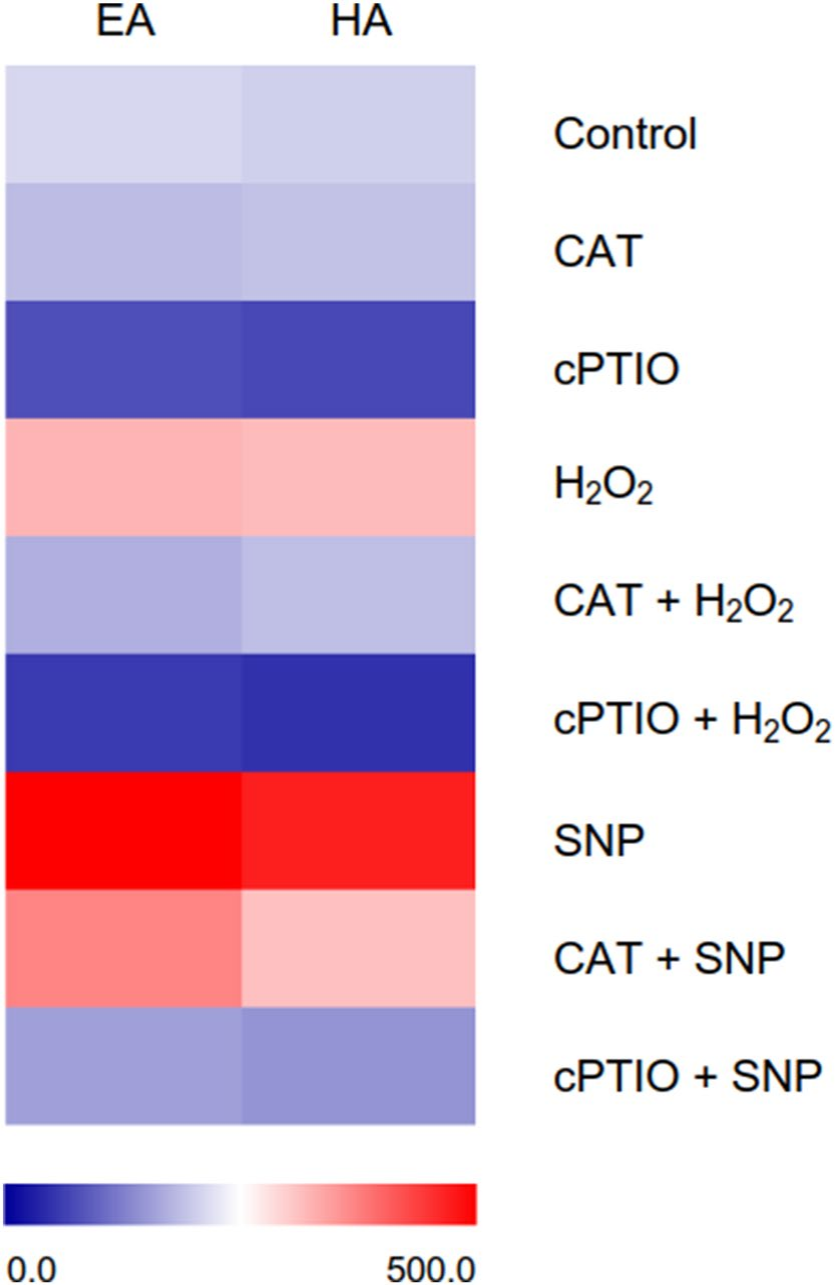
HPLC profiles (UV 460 nm) in different treatments. A color scale indicates the compounds yield, from lowest (blue) to highest (red).

The pigment components in all treatments were analyzed by HPLC, and two substances with the highest absorption peaks were detected: EA and HA. As shown in Fig. 5, H2O2 and SNP treatments significantly increased the production of EA and HA compared with the control group whereas CAT and cPTIO treatment significantly inhibited the yield of EA and HA. In CAT + SNP treatments, CAT could not significantly inhibit the increase of EA and HA production induced by SNP, while in cPTIO + H_2_O_2_ treatment, cPTIO could significantly inhibit the increase of EA and HA yield induced by H_2_O_2_. CAT + H_2_O_2_ and cPTIO + SNP treatment groups restored the yield of EA and HA on the basis of scavengers. The results are consistent with the results of PQs content above (Fig. 4). Collectively, these results indicated that HA and EA are the main PQs production which were promoted by H_2_O_2_ and NO in *S. bambusicola*.

**Figure 6:**
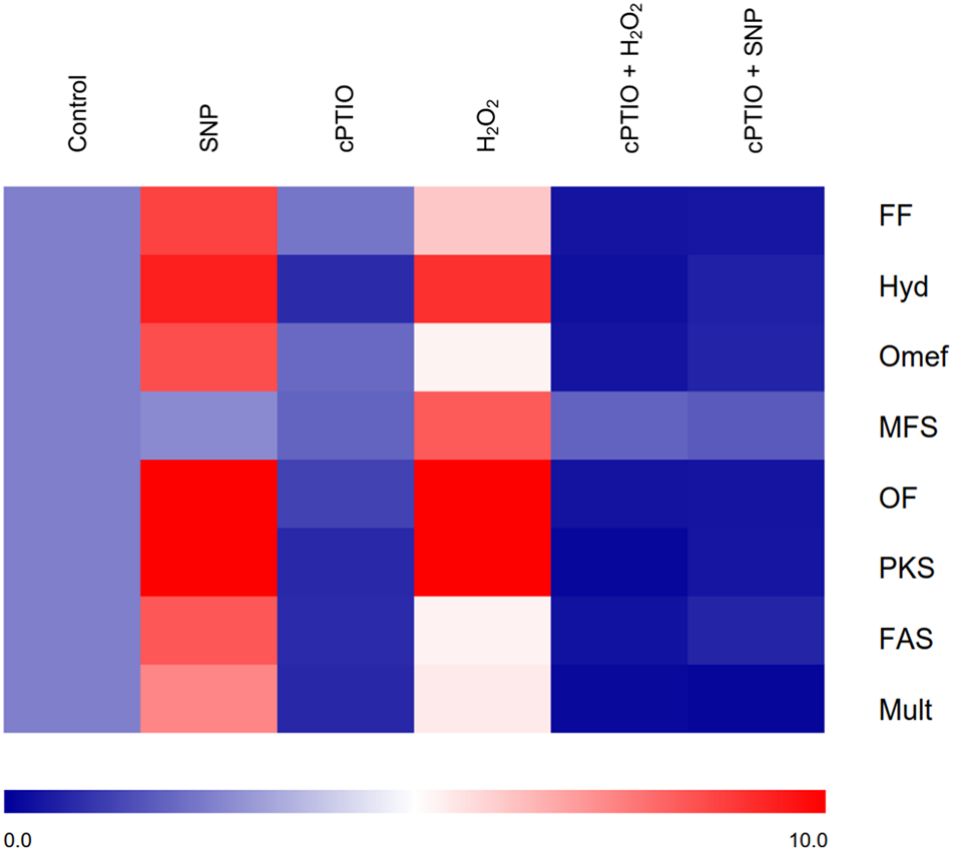
The transcriptional activities of HA biosynthetic genes in different treatments. FF: FAD/FMN-dependent oxidoreductase, Hyd: Hydroxylase, Omef: O-methyltransferase, MFS: Major facilitator superfamily (MFS) transporter, OF: O-methyltransferase/FAD-dependent monooxygenase, PKS: Polyketide synthase, FAS: Fasciclin, Mult: Multicopper oxidase. The transcriptional level of control group was given an arbitrary value of 1. A color scale indicates the transcriptional activities of the different genes, from lowest (blue) to highest (red).

The transcriptional level of HA biosynthetic genes (the genes encoding FF, Hydroxylase (Hyd), Omef, Major facilitator superfamily (MFS) transporter, OF, PKS, Fasciclin (Fas) and Multicopper oxidase (Mult)) in different treatment groups were analyzed by qRT-PCR (Fig. 6). The results showed that the transcriptional level of SNP and H_2_O_2_ treatment groups was higher than that of the control group, which was consistent with the result of PQs content above (Fig. 4), whereas the results in treatments of cPTIO, cPTIO + H_2_O_2_ and cPTIO + SNP were on the contrary. In SNP treatment, the genes encoding PKS (50.9-fold), OF (13.6-fold) and Hyd (9.1-fold) were significantly upregulated as well as in H_2_O_2_ treatment (18.3-fold, 15.3-fold and 8.5-fold). In cPTIO treatment, the genes encoding Mult, PKS and Fas had the strongest transcriptional suppression. In cPTIO + H_2_O_2_ treatment, the transcriptional levels of genes encoding PKS, Mult and Hyd were decreased. In cPTIO + SNP, the transcriptional suppression of genes encoding Mult, OF and PKS was greater than that of other genes. These results showed that H_2_O_2_ and NO significantly enhanced the transcriptional level of HA biosynthetic genes. However, the addition of scavengers significantly inhibited the transcriptional level of HA biosynthetic genes, among which cPTIO and cPTIO + H_2_O_2_ treatment groups significantly reduced HA production. These results indicated that H_2_O_2_ and NO increase the HA yield mainly by promoting the transcriptional level of HA biosynthetic genes.

## Discussion

It is well known that microorganisms can produce a variety of secondary metabolites to enhance their competitiveness, including improving nutrient availability, protecting itself from environmental pressure, decreasing the fitness of their hosts and acting as a metabolic defense mechanism^35^. Recently, more attention has been paid to the study of biosynthesis of secondary metabolites and its regulatory mechanisms in fungi. *S. bambusicola*, a ascomycete with bioactive secondary metabolites, has received much attention due to its commercial value^36^. At present, the signal pathways involved in the biosynthesis of PQs in *S. bambusicola* are not well understood. Therefore, the investigation of signaling pathways of secondary metabolites is essential for foundational and applied research in *S. bambusicola*. In this study, we obtained evidence for the involvement of NO in H_2_O_2_-induced PQs biosynthesis. These results provide insight into the potential mechanism of secondary metabolisms biosynthesis through signal transduction pathways.

Many studies on the regulation of various physiological and developmental processes of plants by H_2_O_2_ had been carried out, but fewer studies were on the regulation of secondary metabolisms of fungi. Deng et al. reported that hypocrellin production was improved by nearly 27% and 25% after 72 h incubation with 10 mM and 20 mM H_2_O_2_, respectively^25^. Herein, we found that H_2_O_2_ can participate in the biosynthesis of PQs by exogenous addition. The production of PQs can be significantly increased by using 0.01 mM H_2_O_2_, and the growth of *S. bambusicola* can be significantly inhibited by high concentration of H_2_O_2_. It likely reflecting that the overload of H_2_O_2_ caused the self-protection mechanism, and the higher dose significantly inhibited the liquid fermentation of *S. bambusicola*, causing damage to the cells and reducing the amount of mycelium. NO has different effects on the production of secondary metabolites in different species. Liu et al. showed that the increase in NO levels alleviates heat stress-induced ganoderic acid accumulation, while the cross-promotion between NO and Ca^2+^ signals is involved in the regulation of heat stress-induced ganoderic acid biosynthesis in *Ganoderma lucidum*^30^. In this study, NO was able to significantly increase the PQs production as a signal molecule. It is speculated that NO may regulate the PQs biosynthetic genes through a series of complex signal transduction mechanisms, but the specific regulation mechanism remains to be studied.

**Figure 7:**
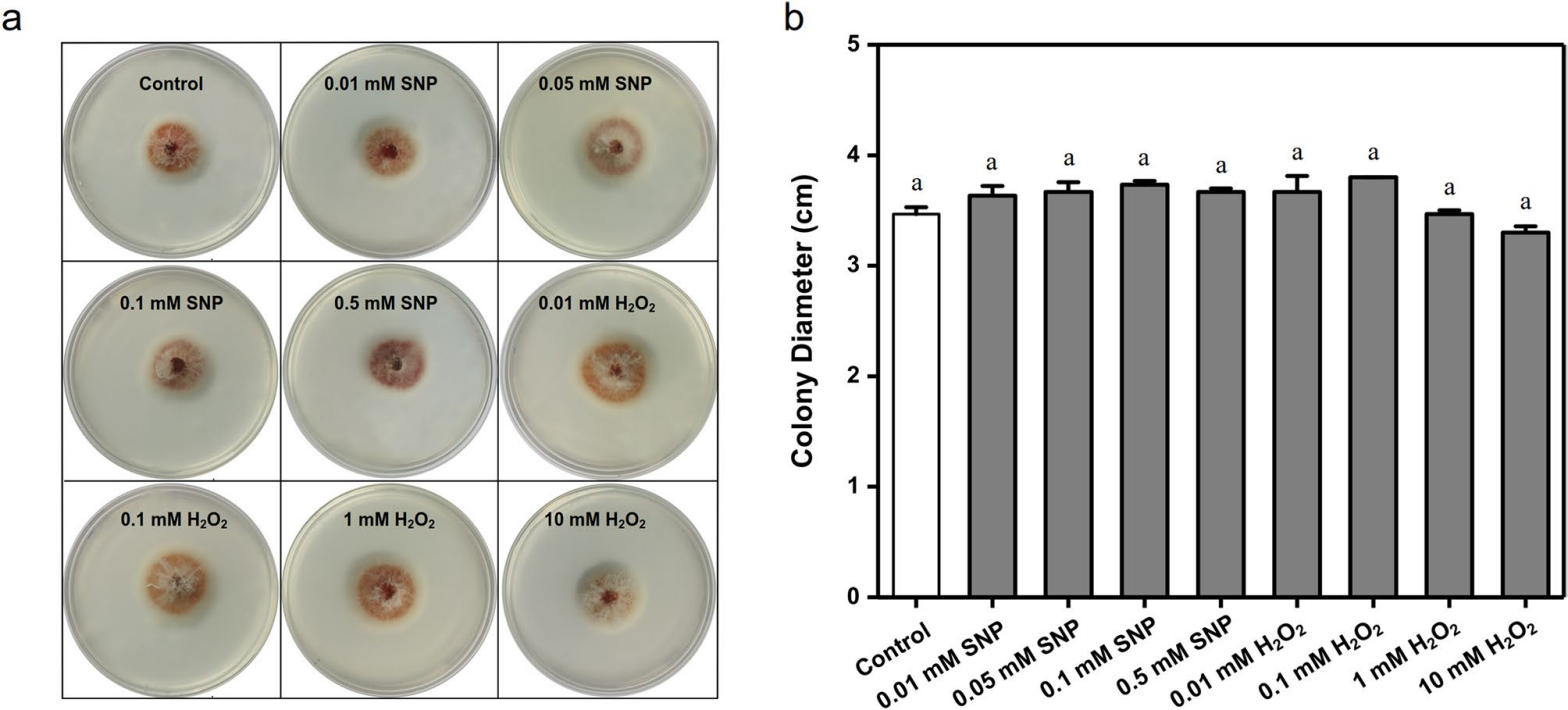
Colonies morphology and diameters of *Shiraia bambusicola* S4201 on different treatments. (**a**) Colonies morphology; (**b**) Diameter sizes. Values are the means of five independent experiments ± SE. Different letters on the histogram indicate significant differences between treatments (*p* < 0.05).

**Figure 8:**
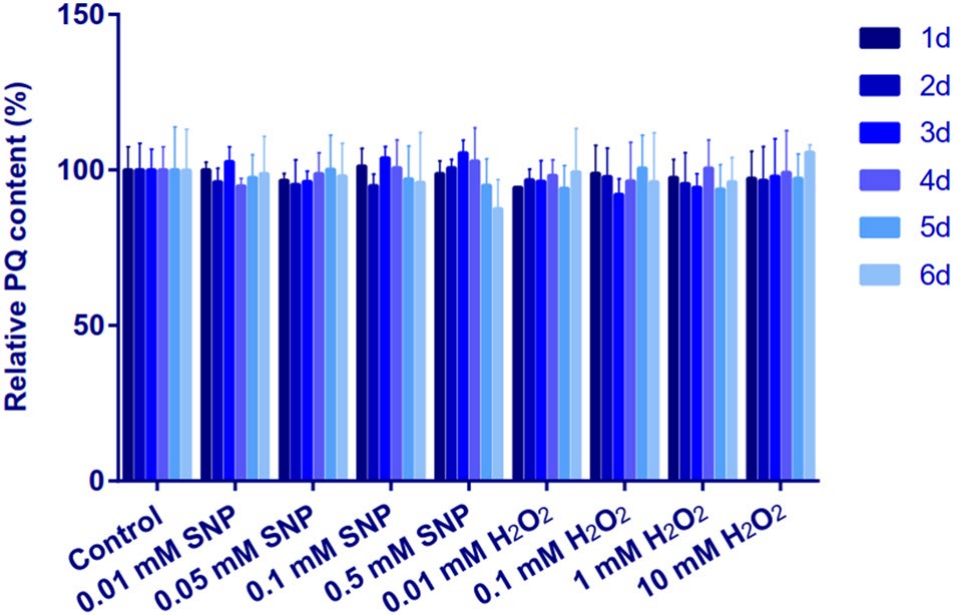
Perylenequinones content on different treatments. Values are the means of five independent experiments ± SE.

As a kind of ROS and reactive nitrogen species (RNS) molecule, H_2_O_2_ and NO may cause oxidative stress as external stress, which will lead to the change of PQs content in *S. bambusicola*. In order to verify whether H_2_O_2_ and NO affect the production of PQs through oxidative stress or chemical contact, the following experiments were carried out. Solid culture of *S. bambusicola* was carried out in dark at 28 °C for 72 h, with different concentrations of H_2_O_2_ and NO, the colony morphology and growth rate were shown in Fig. 7. The colony morphology and diameter size were not significantly different from the control. *S. bambusicola* expressed high tolerance to high concentration of H_2_O_2_ and NO on solid medium. The supernatant of *S. bambusicola* in liquid culture was filtered using sterile nylon membrane, different concentrations of H_2_O_2_ and NO were added respectively and cultured. The content of PQs in different media was detected every 24 h. As shown in Fig. 8, the results showed that there was no significant difference in PQs content. These results proved that H_2_O_2_ and NO did not affect the accumulation of PQs through oxidative stress and chemical contact.

Signal crosstalk between H_2_O_2_ and NO seems to be considered necessary for plants to adapt to complex external conditions and respond to different stresses^37^. H_2_O_2_ may be a cofactor to promote the synthesis of endogenous NO^38^. In this study, H_2_O_2_ treatment can induce an increase of cytosolic NO content. In cPTIO + H_2_O_2_ treatment, cPTIO could significantly inhibit the increase of PQs induced by H_2_O_2_, while in CAT + SNP treatment, CAT could not significantly inhibit the increase of PQs content induced by SNP. These results suggested that H_2_O_2_ and NO signalling pathways are closely related in the progress of increasing PQs yield, and H_2_O_2_ may act as an upstream signal molecule of NO. This is in agreement with the finding that H_2_O_2_ may act upstream of NO production in *P. liquidambari*-induced nodulation and N_2_ fixation^29^. Fungi can use nitrogen sources qualitatively or quantitatively through complex regulatory mechanisms. In order to further verify the role of NO in regulating PQs biosynthesis and speculate the possible mechanism, the following experiments were carried out. Different nitrogen sources such as NaNO_3_, (NH_4_)_2_SO_4_, Gln, NaNO_2_ and no nitrogen sources were added to the culture medium to detect the effect of different nitrogen sources on the yield of PQs. Under the condition of 28 °C and 150 rpm for 80 h, the results showed that the content of PQs in the medium supplemented with NaNO_3_ was higher than that in the medium supplemented with (NH_4_)_2_SO_4_ and Gln, while NaNO_2_ showed toxic effect on the growth of *S. bambusicola*, and the absence of nitrogen source was not conducive to the growth of *S. bambusicola* (Supplementary Fig. S1). It is speculated that compared with (NH_4_)_2_SO_4_ and Gln, NaNO_3_ is more easily metabolized to NO^39^. There are several sites of action of NO in organisms, including catalase, oxyhaemoglobin, iron-sulphur enzymes such as aconitase and NADH dehydrogenase. Nanomolar NO concentrations inactivate or inhibit critical enzymes, including aconitase, affecting the tricarboxylic acid (TCA) cycle^40^. The previous RNA-Seq data showed that the gene encoding citrate synthase was down-regulated while the gene encoding ATP-citrate lyase was up-regulated in the high-yielding HA strain^33^. It catalyzes the decomposition of citric acid to acetyl CoA and oxaloacetic acid, and acetyl CoA is the precursor of PQs biosynthesis. Therefore, we speculate that NO may affect the TCA cycle by binding to aconitase, resulting in the release of more precursors of PQs biosynthesis.

In order to explore whether H_2_O_2_ and NO are involved in the transcriptional regulation of genes related to HA biosynthesis, the transcriptional levels of several related genes were detected, such as PKS, OF and Hyd. The results showed that the transcriptional levels of these genes were significantly up-regulated in H_2_O_2_ and NO-induced strains, and were significantly higher than other HA biosynthetic genes. On the contrary, the addition of scavengers inhibited the transcriptional level. Deng et al. reported that the gene that encoding PKS is essential in HA biosynthesis^41^. Li et al. successfully obtained the HA high-producing strains by overexpressing O-methyltransferase/FAD-dependent monooxygenase gene and the hydroxylase gene^36^. It is suggested that H_2_O_2_ and NO promote HA biosynthesis mainly by regulating the transcription of these three key genes, but the mechanism remains to be further studied. HA and EA have similar structures, and we suspected that their biosynthetic genes are similar. H_2_O_2_ and NO also can increase the yield of EA by increasing the transcriptional level of these three genes.

Although we believe that NO participates in the biosynthesis and accumulation of PQs induced by H_2_O_2_ and promotes the production of EA and HA, it still needs to be further confirmed by gene knockout of genes related to NO synthase and to explore the molecular regulation mechanism of signal molecules to enhance the production of secondary metabolites. In this study, exogenous addition of H_2_O_2_ and NO can effectively improve the ability of *S. bambusicola* to produce PQs (Supplementary Fig. S2). Therefore, we speculate whether the activation of endogenous H_2_O_2_ and NO signals by physical or chemical methods is also conducive to the accumulation of secondary metabolites of *S. bambusicola* such as EA and HA in fermentation production. However, there are many uncertain factors in fermentation production, and its effect still needs to be further verified.

## Supplementary Information

Supplementary Information.
