# Supplementary material for: Nitric oxide regulates perylenequinones biosynthesis in Shiraia bambusicola S4201 induced by hydrogen peroxide
Source: Sci Rep. 2021 Jan 27;11:2365. doi: 10.1038/s41598-021-81990-2 (PMC7840948; doi:10.1038/s41598-021-81990-2)
Supplement: Supplementary file 1 — Supplementary Information. [file 41598_2021_81990_MOESM1_ESM.docx]

**Supplementary Information**

**Journal:** Scientific Reports

**Manuscript:**Nitric Oxide Regulates Perylenequinones Biosynthesis in *Shiraia bambusicola* S4201 Induced by Hydrogen Peroxide

Ning Zhao†, Yingying Yu†, Yunxia Yue, Mingzhu Dou, Bingjing Guo, Shuzhen Yan, Shuanglin Chen*

College of Life Sciences, Nanjing Normal University, Nanjing 210023, China

* Corresponding author

E-mail: chenshuanglin@njnu.edu.cn (SLC)

Tel.: +86-25-8589-1050

Address: No.1 Wenyuan Road, Qixia District, Nanjing, P. R. China

†These authors contributed equally to this work.

Supplementary Table

| **Table S1** List of primer sequences designed for qRT-PCR | | |
| --- | --- | --- |
| Gene | Sense primer | Antisense primer |
| GAPDH | ACGATGCGACCAATACGA | AACTCTTCCTCCTACACTTCAG |
| FAD/FMN-dependent oxidoreductase | CGGGCGATGCGAAGTTGC | AGTAGGTGGCGTTGGTGTGG |
| Hydroxylase | CACTGCGGTCACTTTGCGTAAC | GAGATGCTGGGCGGGCTTG |
| O-methyltransferase | ATGTAGTAGATGGAGGTT | TTAGTTGGGGATATTTGC |
| Major facilitator superfamily (MFS) transporter | AGCAGTCATAGTCTTCTT | CATACAGGTCCATCTCAT |
| O-methyltransferase/  FAD-dependent monooxygenase | CAAACTCTCGCCCAAACTGTATC | GCTTCCTGTGCCGTCTCG |
| Polyketide synthase | TCAGACGCACTCACAATAACC | GGACCAAAGCACAACAAAGC |
| Fasciclin  Multicopper oxidase | CACACTATCGTTGGAGAA  CAGTGGTAGAAGCAGAGAA | GATGTTGTCGTAGCCTTT  AATGTGGCAATGGAATAGC |

Supplementary Figures


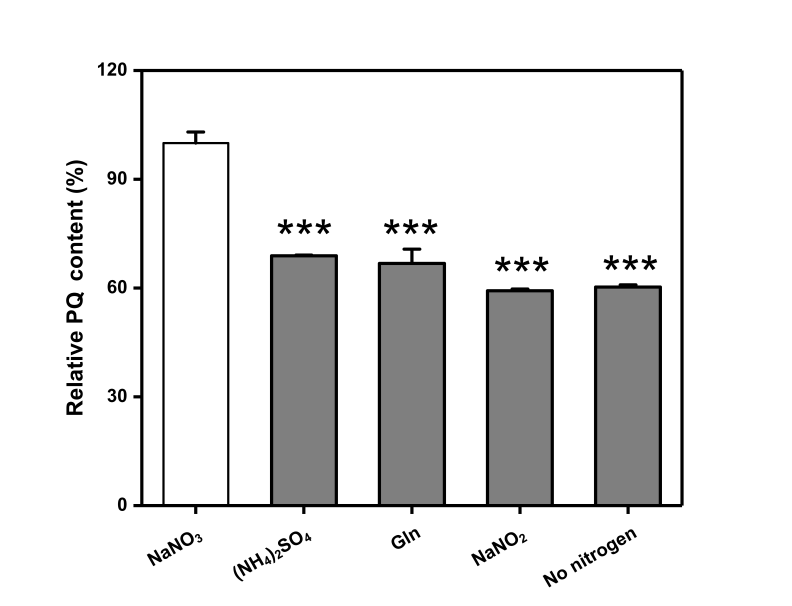


**Figure S1.** Effects of different nitrogen sources on perylenequinones production.

**
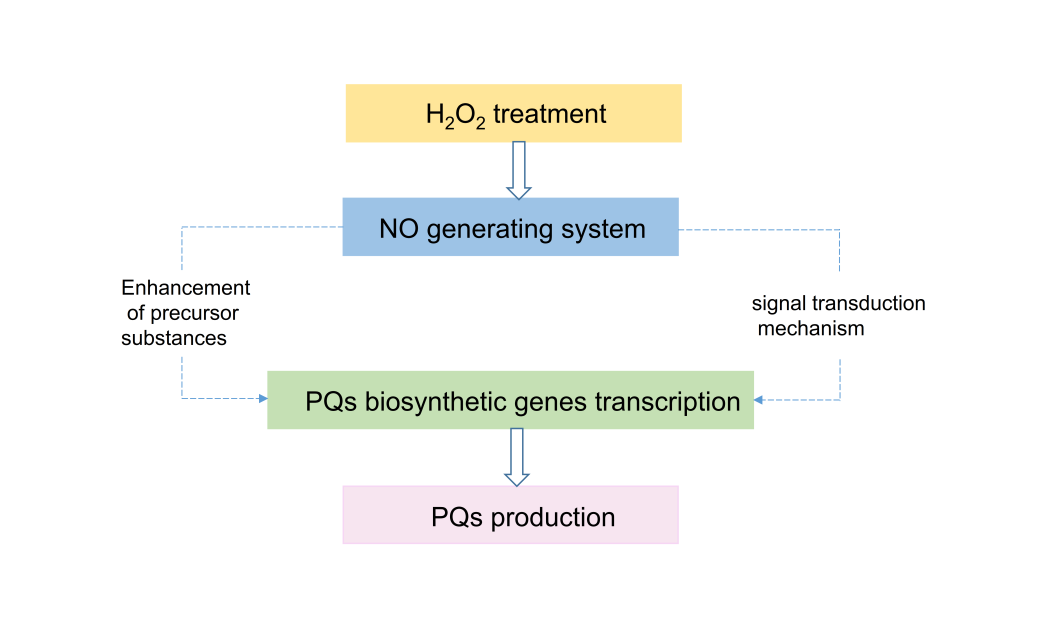
**

**Figure S2.** Schematic representation of a hypothetical model of H_2_O_2_ and NO signalenhance perylenequinones production.
